# S-Nitrosation of *Arabidopsis thaliana* Protein Tyrosine Phosphatase 1 Prevents Its Irreversible Oxidation by Hydrogen Peroxide

**DOI:** 10.3389/fpls.2022.807249

**Published:** 2022-02-11

**Authors:** Valérie Nicolas-Francès, Jordan Rossi, Claire Rosnoblet, Carole Pichereaux, Siham Hichami, Jeremy Astier, Agnès Klinguer, David Wendehenne, Angélique Besson-Bard

**Affiliations:** ^1^Agroécologie, CNRS, INRAE, Institut Agro, Université de Bourgogne, Université Bourgogne Franche-Comté, Dijon, France; ^2^Fédération de Recherche (FR3450), Agrobiosciences, Interactions et Biodiversité (FRAIB), CNRS, Toulouse, France; ^3^Institut de Pharmacologie et de Biologie Structurale (IPBS), Université de Toulouse UPS, CNRS, Toulouse, France

**Keywords:** *Arabidopsis thaliana*, protein tyrosine phosphatase 1, nitric oxide, S-nitrosation, H_2_O_2_, oxidation, post-translational modifications, mitogen-activated protein kinases

## Abstract

Tyrosine-specific protein tyrosine phosphatases (Tyr-specific PTPases) are key signaling enzymes catalyzing the removal of the phosphate group from phosphorylated tyrosine residues on target proteins. This post-translational modification notably allows the regulation of mitogen-activated protein kinase (MAPK) cascades during defense reactions. *Arabidopsis thaliana* protein tyrosine phosphatase 1 (*At*PTP1), the only Tyr-specific PTPase present in this plant, acts as a repressor of H_2_O_2_ production and regulates the activity of MPK3/MPK6 MAPKs by direct dephosphorylation. Here, we report that recombinant histidine (His)-*At*PTP1 protein activity is directly inhibited by H_2_O_2_ and nitric oxide (NO) exogenous treatments. The effects of NO are exerted by S-nitrosation, i.e., the formation of a covalent bond between NO and a reduced cysteine residue. This post-translational modification targets the catalytic cysteine C265 and could protect the *At*PTP1 protein from its irreversible oxidation by H_2_O_2_. This mechanism of protection could be a conserved mechanism in plant PTPases.

## Introduction

Protein phosphatases and protein kinases are key enzymes that regulate many biological processes by protein dephosphorylation or phosphorylation, respectively. In humans, 197 protein phosphatases were identified. This number is quite comparable to that of *Arabidopsis thaliana*, as 150 protein phosphatases have already been identified (Chen et al., 2017; Bheri et al., 2021). In Eukaryotes, protein phosphatases are classified in two major categories according to the amino acid target residue(s) of the dephosphorylation process: protein tyrosine (Tyr) phosphatases (PTPases; EC 3.1.3.48) and phosphoprotein serine/threonine (Ser/Thr) phosphatases (PPases). PTPases are themselves divided into two subgroups: tyrosine-specific protein tyrosine phosphatases (Tyr-specific PTPases), which include intracellular PTPases and receptor-like PTPases, and dual-specificity PTPases that constitute a particular subgroup of PTPases since they are able to hydrolyze both phosphoserine/phosphothreonine and phosphotyrosine residues (Luan, 2003). All PTPases possess a signature motif of 11 amino acids at the active site (I/V)H**C**XAGXXR(S/T)G, which contains the cysteine (Cys) catalytic residue (indicated in bold) involved in the formation of a phosphoenzyme intermediate during the reaction (Guan and Dixon, 1991). Tyr phosphorylation and consequently Tyr dephosphorylation have recently emerged as important mechanisms for transmembrane signaling in plants, in particular in immunity processes (Mühlenbeck et al., 2021).

The *Arabidopsis thaliana* protein Tyr phosphatase 1 (*At*PTP1), an intracellular PTPase, was the first PTPase characterized in higher plants. Like most of the other plant species, there is only one Tyr-specific protein phosphatase in *A. thaliana* (Kerk et al., 2008). Its PTPase activity was measured *in vitro* by Xu et al. (1998). This protein of 340 amino acids and 37.8 kDa is located in both the cytosol and the nucleus (Bartels et al., 2009). The *At*PTP1 gene is preferentially expressed in roots, stems, and flowers. It is less expressed in leaves (Xu et al., 1998; data of gene expression map of Arabidopsis development from Schmid et al., 2005 using eFP Browser at bar.utoronto.ca; Winter et al., 2007). PTPases play important roles in the regulation of mitogen-activated protein kinases (MAPKs) cascades (Ichimura et al., 2002). In particular, *At*PTP1 is known to dephosphorylate *At*MPK6 (Gupta and Luan, 2003) and *At*MPK4 (Huang et al., 2000) *in vitro*. In addition, it can interact *in vivo* with *At*MPK3 and *At*MPK6 (Bartels et al., 2009; Zhou et al., 2017; Zhao et al., 2020). Thus, *At*PTP1 could inhibit defense reactions through MAPK inactivation by dephosphorylation and also through suppression of salicylic acid (SA) synthesis in association with *At*MKP1 (MAPK Phosphatase 1), a dual-specificity PTPase whose role is partially redundant to that of *At*PTP1 (Bartels et al., 2009). Recent data suggest that *At*PTP1 and *At*MKP1 negatively regulate H_2_O_2_ production by the NADPH oxidase *AtRbohD* activity and inhibit the activity levels of *At*MPK3 and *At*MPK6 during defense responses to *Verticillium dahlia* toxins (Zhao et al., 2020). Conversely, *At*PTP1 is also reversibly inactivated *in vitro* by H_2_O_2_ treatment (Gupta and Luan, 2003).

*At*PTP1 is regulated both at the transcriptional and post-translational levels. Indeed, *At*PTP1 transcript expression is induced in response to salt stress (Xu et al., 1998; Zhou et al., 2017) and repressed in response to cold (Xu et al., 1998). Recently, it was reported that in poplar, the overexpression of the Tyr-specific protein phosphatase-encoding gene *PdPTP1* led to an increase of the plant sensitivity to salt stress. This process was linked to increased levels of ROS and perturbations in Na^+^ and K^+^ ion homeostasis. *Pd*PTP1 was also shown to interact with PdMPK3 and PdMPK6 *in vitro* and *in vivo* (Lu et al., 2020). Thus, it appears that the PTP1 protein has an important role in the response to salt stress in plants. In response to submergence, *At*PTP1 is regulated at the post-translational level by *At*SnRK1 (Sucrose non-fermenting-1-related protein kinase) through the phosphorylation of its threonine (Thr) 6, Ser 7, and Ser 8 residues. Phosphorylation of *At*PTP1 would prevent its interaction with *At*MPK6, which would consequently contribute to the enhancement of *At*MPK6 signaling (Cho et al., 2016).

The protein most closely related to *At*PTP1 in humans is the Tyr-specific protein phosphatase PTP1B. PTP1B has been extensively studied in humans because of its involvement in the insulin signaling pathway and in cell proliferation. *At*PTP1 and human PTP1B share 35% identity between their amino acid sequences but the catalytic site of the two proteins, as all PTPases, is very well conserved.

Regarding the regulation of PTP1B activity, different mechanisms involving post-translational modifications have been characterized. PTP1B can be regulated by acetylation, phosphorylation, sulfhydration, S-nitrosation, and several levels of oxidation, which lead to reversible or irreversible modifications. PTP1B was shown to by acetylated at methionine (Met) 1 residue (Charbonneau et al., 1989) while phosphorylation occurs mostly at the Ser 50 residue (Moeslein et al., 1999; Ravichandran et al., 2001; Zhou et al., 2013) even if other Ser and some Tyr and Thr residues can be targeted (Flint et al., 1993; Liu and Chernoff, 1997; Moeslein et al., 1999; Rush et al., 2005; Zhou et al., 2013). Phosphorylation leads to activation or inhibition of PTP1B activity *in vitro* (Moeslein et al., 1999) and *in vivo* (Moeslein et al., 1999; Ravichandran et al., 2001). Both oxidation and S-nitrosation of PTP1B target the catalytic Cys residue C215. The PTPase signature motif gives to the catalytic Cys a low p*Ka*, which increases its nucleophilicity and makes the protein particularly susceptible to inactivation by reactive oxygen species like H_2_O_2_ and superoxide (Denu and Tanner, 1998). Indeed, the catalytic Cys residue of PTPases has an unusual p*Ka* between 4.5 and 5.5 when it is typically around 8.5. This feature allows the catalytic Cys to be under the thiolate anion form at neutral pH (Zhang and Dixon, 1993; Lohse et al., 1997). This low p*Ka* value thus makes the Cys very sensitive to redox-dependent regulation (Salmeen and Barford, 2005). Reversible oxidation of the catalytic Cys of PTPase proteins induces a transient inhibition of their activity, which suppresses the dephosphorylation of their target proteins. The oxidation of PTP1B enzyme in solution was studied by X-ray crystallography and mass spectrometry (MS) after a treatment with H_2_O_2_. This treatment initially leads to the formation of a sulfenic acid intermediate (PTP1B-SOH), which is rapidly converted into sulphenyl-amide species. PTP1B then undergoes major conformational changes in its catalytic site that inhibit substrate binding. The formation of sulphenyl-amide is an unusual modification that protects the catalytic Cys from irreversible oxidation and promotes its reversible reduction by thiols, which allows the reactivation of PTP1B activity. This modification is reversible but after a prolonged exposure to high concentrations of H_2_O_2_, sulfenic acid is then transformed into sulfinic acid (PTP1B-SO_2_H) and sulfonic acid (PTP1B-SO_3_H), which are irreversibly oxidized forms of the enzyme (Salmeen et al., 2003; van Montfort et al., 2003). *In vivo*, PTP1B is reversibly inhibited by oxidation in cells following stimulation by insulin (Mahadev et al., 2001) and by the epidermal growth factor (Lee et al., 1998). Londhe et al. (2020) demonstrated the existence of a molecular interaction between the reversible oxidized form of PTP1B and the 14-3-3ζ proteins. Destabilization of this interaction prevents inactivation of PTP1B by reactive oxygen species and decreases phosphorylation of the epidermal growth factor receptor. It has been demonstrated *in vitro* that PTP1B is S-nitrosable by two NO donors, S-nitroso-N-acetylpenicillamine (SNAP) and diethylamine NONOate (DEA-NONOate), as well as by the trans-nitrosylating agent S-nitrosoglutathione (GSNO), on its Cys residue C215 but also C32 and C92, these last two being located at the protein surface (Chen et al., 2008). PTP1B was also found to be S-nitrosated *in vivo* (Li and Whorton, 2003; Hsu et al., 2016). S-nitrosation of PTP1B reduces its activity *in vitro* and *in vivo* (Li and Whorton, 2003). Interestingly, it has been shown *in vitro* that S-nitrosation of PTP1B may have a protective effect against protein inactivation by oxidation. Indeed, treatment of PTP1B with relatively high concentrations of H_2_O_2_ leads to a modification of Cys 215 into -SO_2_H/-SO_3_H but does not occur if PTP1B is previously treated by SNAP (Salmeen et al., 2003). In addition, Chen et al. (2008) found by mass spectrometry that pre-treatment of PTP1B with a NO donor prevents modification of the enzyme by H_2_O_2_. These data indicate that S-nitrosation would protect the enzyme from H_2_O_2_ irreversible oxidation. The prevention of irreversible oxidation and inactivation of PTP1B could also be provided by reversible sulfhydration of C215 by H_2_S, which was shown to inactivate the protein *in vitro* and in HeLa cells. This mechanism would regulate the endoplasmic reticulum (ER) stress response (Krishnan et al., 2011).

A different regulatory mechanism has been identified for the soybean *Gm*PTP protein under oxidizing conditions (Dixon et al., 2005). *Gm*PTP was shown to be relatively insensitive to inactivation by H_2_O_2_, unlike its mammalian counterparts such as PTP1B, but was hypersensitive to S-glutathionylation in the presence of oxidized glutathione (GSSG). More precisely, contrary to the catalytic Cys 266 (C266) residue, Cys 78 (C78) and Cys 176 (C176), which are activity regulators, are direct targets of S-glutathionylation as this modification inactivates the enzyme. The authors of this study hypothesized a chain reaction. First, C176 would undergo rapid thiolation by GSSG, which would inactivate *Gm*PTP. Secondly, C78, which is close to the active site, could be S-glutathionylated but less easily than C176. Finally, thiolated C78 would be able to undergo a disulfide exchange with the catalytic C266, thus releasing GSH to form an intramolecular C78-C266 disulfide or a disulfide-linked dimer of the protein. This mechanism would constitute a double protection of the catalytic C266 by blocking this residue in a basal state in the form of sulfenyl-amide, as described for PTP1B (Salmeen et al., 2003; van Montfort et al., 2003), and which could be reactivated rapidly following reduction by thiols.

S-nitrosation of *At*PTP1 has never been studied. Here, after producing and purifying a recombinant histidine (His)-*At*PTP1 protein, we verified by mass spectrometry its S-nitrosation after application of the biotin switch method. We then tested the effects of exogenous NO or H_2_O_2_ on its activity. We found that similarly to human PTP1B, the activity of *At*PTP1 is inhibited by S-nitrosation and that this post-translational modification probably would allow its protection against irreversible oxidation *in vivo*. The protection of the catalytic Cys residue by NO *via* S-nitrosation against irreversible oxidation could be a conserved mechanism in plant PTPases.

## Materials and Methods

### Plasmid Construction

The coding sequence of *AtPTP1* (*At1g71860*) was amplified by PCR from *A. thaliana* leaf cDNA using Phusion Taq Polymerase and then cloned into the PCR8 vector (Thermofisher). The coding sequence of *AtPTP1* was then transferred into the pHGWA vector (Busso et al., 2005) by LR recombination, resulting in pHGWA-*AtPTP1*.

### His-*At*PTP1 Production and Purification

*Arabidopsis thaliana protein tyrosine phosphatase 1* cDNA was expressed in *Escherichia coli* Rosetta II bacteria under the control of the T7 RNA polymerase promoter to allow synthesis of a recombinant *At*PTP1 protein with a His-tag at the NH_2_ extremity. Bacteria were grown at 37°C in Luria-Bertani (LB) medium until the absorbance at 600 nm reached .6. Protein production was then induced by .5 mM isopropyl 1-thio-β-D-galactopyranoside (IPTG) overnight at 25°C. Bacterial cells were then lysed in lysis buffer [50 mM Tris–HCl pH 7.5, 300 mM NaCl, 1% (v/v) triton X100, Protease Inhibitor Cocktail (Roche), 2.5 mg·ml^−1^ lyzozyme, 21 mg·ml^−1^ DNAse, and 6.25 mg·ml^−1^ RNAse], briefly sonicated and centrifuged at 20,000*g* for 30 min. The soluble proteins were passed through .22 μm filters prior to purification of His-*At*PTP1 by Ni^2+^ affinity chromatography using a His-TRAP™ column by FPLC on ÄKTA™ Purifier, according to the manufacturer’s instructions (GE Healthcare). After selection of fractions containing His-*At*PTP1, these were pooled and dialyzed against 25 mM HEPES-KOH pH 7.7, 1 mM ethylenedinitrilotetraacetic acid (EDTA), and 500 mM NaCl buffer. Protein concentration was assessed by absorbance reading at 280 nm using a NanoDrop^TM^ spectrophotometer (ThermoScientific). His-*At*PTP1 was stored at −20°C in 20% glycerol without loss of activity.

### Biotin Switch Assay

The biotin switch assay was performed as described by Jaffrey and Snyder (2001) with some changes described in Astier et al. (2012). Briefly, 300 μg of His-*At*PTP1 were mixed with 300 μl of HEN buffer {25 mM HEPES-NaOH pH 7.7, 1 mM EDTA, .1 mM neocuproine, and .5% 3-[(3-cholamidopropyl)dimethylammonio]-1-propanesulfonate (CHAPS)} containing either GSH or GSNO, which were used at a final concentration of 100 μM. The incubation was done at 25°C overnight in the dark. After removing GSH/GSNO by acetone precipitation, the pellet was resuspended in 300 μl blocking buffer [20 mM methylmethane thiosulfonate (MMTS) and 2% sodium docecyl sulfate (SDS) in HEN buffer] and was incubated 20 min at 50°C with shaking (350 rpm). MMTS was removed by acetone precipitation and the pellet was resuspended in 300 μl HENS (HEN with 1% SDS) with 1 mM N-[6-(biotinamido)hexyl]-3-(2-pyridyldithio) propionamide (biotin-HPDP; ThermoFisher Scientific) and 50 mM ascorbate. After 1 h at room temperature, the proteins were precipitated by acetone and resuspended in 100 μl of 25 mM ammonium bicarbonate (AMBIC). Proteins were digested overnight at 37°C with 6 μg of trypsin (Promega). The samples were then lyophilized and analyzed by mass spectrometry.

### Mass Spectrometry Analysis

The peptide mixtures were analyzed by nano liquid chromatography tandem mass spectrometry (nanoLC-ESI-MS/MS) using an Orbitrap Velos mass spectrometer (ThermoFisher Scientific). Peptides were eluted on an in-house C18 reverse-phase microcolumn, 75 μm ID × 50 cm packed with Reprosil-Pur C18-AQ 3 μm resin (Dr Maisch GmbH; Ammerbuch, Germany) and analyzed by high-resolution MS, followed by tandem MS/MS of the 20 most abundant ions.

### Bioinformatics Analysis

Acquired MS and MS/MS data were searched with Mascot (version 2.7.0, http://matrixscience.com) against a custom-made database containing PTP1 Arabidopsis sequence and all K12 *E. coli* entries from the UniProtKB database (*E. coli* K12_UniprotSP_20180430.fasta; 5,979 sequences; and 1,850,060 residues). The search included methionine oxidation and N-Ter acetylation. Regarding possible Cys residue modifications, we searched for biotin-HPDP addition, glutathionylation (GSH addition), or methylthiolation (-S-CH_3_) due to MMTS treatment. Trypsin/P was chosen as the enzyme and two missed cleavages were allowed. The mass tolerance was set to 5 ppm for the precursor ion and to .8 Da for fragment ions. Validation of identifications was performed through a false-discovery rate set to 1% at protein and peptide-sequence match level, determined by target-decoy search using the Proline software version 1.6.^1^ Raw MS signal extraction of identified peptides was performed with Proline across different samples. For all comparison, statistical analysis by ANOVA one way was applied to the abundance values.

### Treatment of His-*At*PTP1 With GSH/GSNO/SNAP or H_2_O_2_ Before the Measurement of Its Activity

To study the effect of its S-nitrosation, the His-*At*PTP1 protein was treated overnight at 25°C in HEN buffer with GSH or GSNO (100 or 500 μM), or SNAP (250 µM, 500 µM, 750 µM or 1 mM). These molecules were then removed by passing the protein samples through G50 exclusion columns (Micro Bio-Spin™ P-6 Gel Columns; Biorad), which allow the elimination of molecules whose molecular weight is lower than 6,000 Da and then the extract was treated or not with 20 mM DTT for 30 min at 25°C.

To study the effect of its oxidation, the His-*At*PTP1 protein was treated for 30 min at 25°C with 50 µM, 100 µM, 250 µM, 500 µM, 750 µM, 1 mM, 2 mM or 5 mM H_2_O_2_. The H_2_O_2_ molecules were then removed by treatment with 10 μg of catalase for 30 min and then the extract was treated or not with 20 mM DTT for 30 min at 25°C.

### *In vitro* His-*At*PTP1 Enzymatic Assays

His-*At*PTP1 enzyme activity assay was performed at 25°C using *p*-nitrophenyl phosphate (*p*NPP disodium salt, Sigma Aldrich) that is hydrolyzed to *p*-nitrophenol (*p*NP). *p*NP production was monitored by absorbance at 405 nm. The conditions of the assay were those described by Montalibet et al. (2005). Briefly, the reaction was initiated by adding 20 ng of His-*At*PTP1 to 200 μl assay buffer (50 mM BIS-TRIS pH 6.3, 2 mM EDTA, 2% glycerol, .01% triton X100, and 1 mM *p*NPP) in a 96-well plate and mixed vigorously for 1 min. The reaction was stopped at 15, 30, 45, or 60 min later with 20 μl of 10 M NaOH and read at 405 nm on a plate reader (Molecular Devices). To convert the absorbance value at 405 nm to *p*NP concentration, the constant ε.l (949 mol^−1^) was determined using the Beer–Lambert law from a standard range of *p*NP.

### Statistical Analysis

One-way or two-way ANOVA were performed using XLSTAT software (Addinsoft) followed by a multiple comparison procedure with the Fisher’s LSD method.

## Results

### GSNO-Treated His-*At*PTP1 Recombinant Protein Is S-Nitrosated at the Catalytic Cys C265

**Figure 1:**
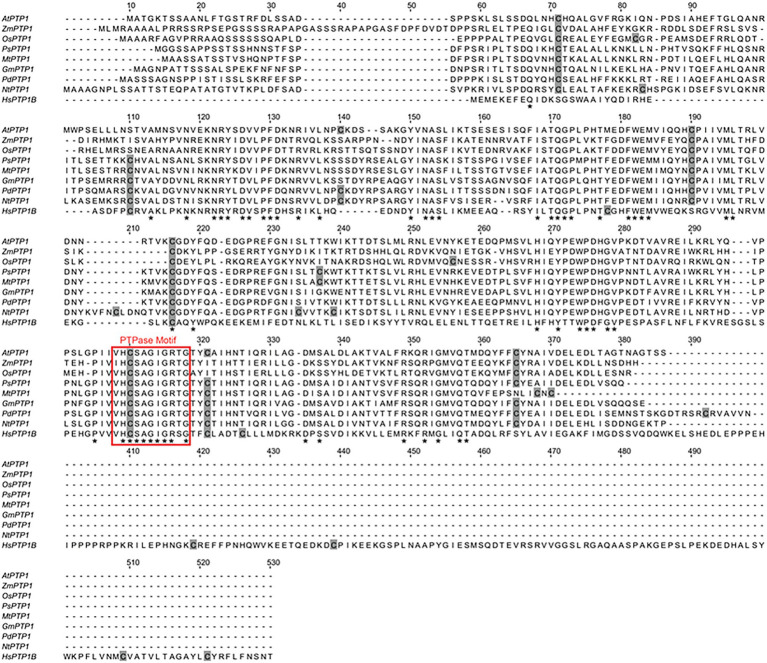
Sequence alignment of eight plant Protein Tyrosine Phosphatase 1 (PTP1) proteins, including *Arabidopsis thaliana* Protein Tyrosine Phosphatase 1 (*At*PTP1), and the human PTP1B protein. Perfectly conserved amino acid residues are indicated by stars. Cys residues are indicated in gray. The typical conserved PTPase signature motif is highlighted by a red frame. The sequences compared are as follows: *Arabidopsis thaliana* *At*PTP1, O82656; *Zea mays* *Zm*PTP1, NP_001149088; *Oryza sativa* *Os*PTP1, Q2QX07; *Pisum sativum Ps*PTP1, O82710; *Medicago truncatula* *Mt*PTP1, A0A072VQ92; *Glycine max* *Gm*PTP1, O82687; *Populus nigra* × (*Populus deltoides* × *P. nigra*) *Pd*PTP1, Lu et al., 2020; *Nicotiana tabacum* *Nt*PTP1, A0A1S4A2F4; and *Homo sapiens* *Hs*PTP1B, P18031.

**Figure 2:**
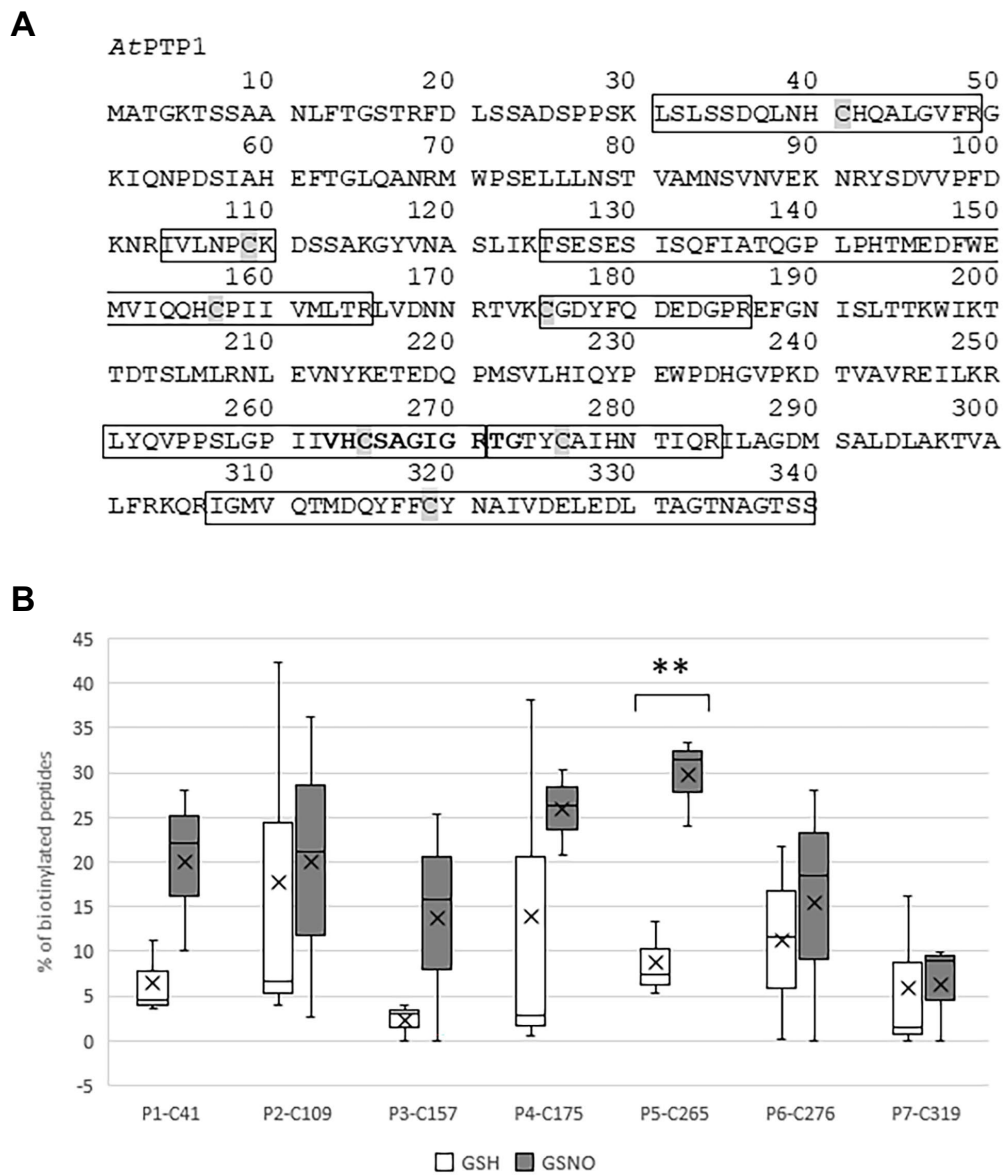
Identification by mass spectrometry of the Cys residues of histidine (His)-*At*PTP1 targeted by S-nitrosation. **(A)** Primary sequence of *At*PTP1 protein. The seven peptides identified by mass spectrometry are boxed, the Cys residues are shaded and the active site signature motif of PTPases is indicated in bold. **(B)** Relative percentage of biotinylated peptide to the total abundance of this peptide in the sample following treatment of His-*At*PTP1 by 100 μM glutathione (GSH) or S-nitrosoglutathione (GSNO). Values are the results of three mass spectrometry analyses. Significant difference is denoted by an asterisk: ^**^p ≤ .01.

*Arabidopsis thaliana* *Protein Tyrosine Phosphatase 1* cDNA encodes a 340 amino acid protein that shares homology with PTP1 proteins from other organisms, such as *Zea mays Zm*PTP1, *Oryza sativa Os*PTP1, *Pisum sativum Ps*PTP1, *Medicago truncatula Mt*PTP1, *Glycine max Gm*PTP1, *Populus nigra* × (*Populus deltoides* × *P. nigra*) *Pd*PTP1, *Nicotiana tabacum Nt*PTP1, or *Homo sapiens Hs*PTP1B (Figure 1). In particular, the critical region for catalysis with the Cys involved in the formation of the phosphoenzyme reaction intermediate is conserved between all of these proteins. In 1998, Xu et al. (1998) confirmed that the recombinant protein GST-*At*PTP1 was a highly active Tyr-specific protein phosphatase. In regard to its key role in signaling, we investigated here whether *At*PTP1 could be modified by S-nitrosation as reported for PTP1B. For this purpose, a recombinant *At*PTP1 protein with a His-tag at the NH_2_ extremity was produced in *E. coli* bacteria and purified by metal-affinity chromatography. Then, 300 μg of recombinant His-*At*PTP1 protein were incubated overnight either with 100 μM GSH or GSNO and subjected to the biotin switch method. This technique consists in replacing the NO groups of nitrosothiols by a biotin molecule after having protected free thiols by alkylation (Jaffrey and Snyder, 2001). After incubation with biotin-HPDP, the His-*At*PTP1 protein samples were suspended in 25 mM AMBIC and digested by trypsin in the absence of a reducing agent to preserve the Cys-biotin bond. The trypsic peptides were identified by LC–MS after interrogation against the *E. coli* K12 library enriched with PTP1 sequences from *A. thaliana*. According to the sequence of the His-*At*PTP1 protein, seven Cys-containing peptides can be generated by tryptic digestion of His-*At*PTP1 (Figure 2A). We named the peptides generated after trypsin cleavage by their order in the protein sequence (from NH_2_ to COOH) and by the number of the Cys contained in the peptide (e.g., the sequence named P5-C265 is the fifth peptide generated by trypsin in the sequence and it contains cysteine C265). LC–MS analysis of GSNO- or GSH-treated His-*At*PTP1 protein after application of the biotin switch method and trypsin digestion allowed the detection of the expected seven peptides. The mass difference (shift) between peptides identified the following Cys modifications: S-methylthiolation due to MMTS protection, S-biotinylation, and S-glutathionylation. The relative abundance of each Cys modification after His-*At*PTP1 treatment either with GSH or GSNO was reported on Figure 2B for S-biotinylation, on Supplementary Figure S1A for S-methylthiolation and on Supplementary Figure S1B for S-glutathionylation. The GSNO-treated His-*At*PTP1 control condition, without the addition of HPDP-biotin, showed that none of the peptides were found to be biotinylated under this condition, thus ensuring that the His-*At*PTP1 protein was not biotinylated during its synthesis in the bacteria and that the observed biotinylation results from the application of the biotin switch method (data not shown). In contrast, methylthiolated and biotinylated forms were detected for all seven Cys-containing peptides. Nevertheless, the presence of biotinylated peptides in the absence of NO donor (in GSH condition) indicates that the protection of free Cys by MMTS was not complete as often described in the literature (Lamotte et al., 2015). GSH treatment resulted in 73%–98% methylthiolation of each Cys. A significant increase in biotinylation was only observed for the P5-C265 peptide when comparing GSH and GSNO treatments (Figure 2B). This modification was correlated to a concomitant decrease in methylthiolation (Supplementary Figure S1A). Other residues (C41 and C157 particularly) could also be targets of S-nitrosation but the data obtained between GSH and GSNO are not statistically different (Figure 2B). A significant increase in the relative percentage of glutathionylation of P4-C175, P6-C276, and P7-319 peptides in response to GSNO compared with the GSH control was also noticed (Supplementary Figure S1B). Since the C265 residue identified as S-nitrosated belongs to the active site of the enzyme, we wondered whether this post-translational modification has a positive or negative effect on the activity of the His-*At*PTP1 enzyme. We sought to answer this question by measuring the enzymatic activity of His-*At*PTP1 *in vitro*.

### S-Nitrosation Inhibits the Activity of His-*At*PTP1

**Figure 3:**
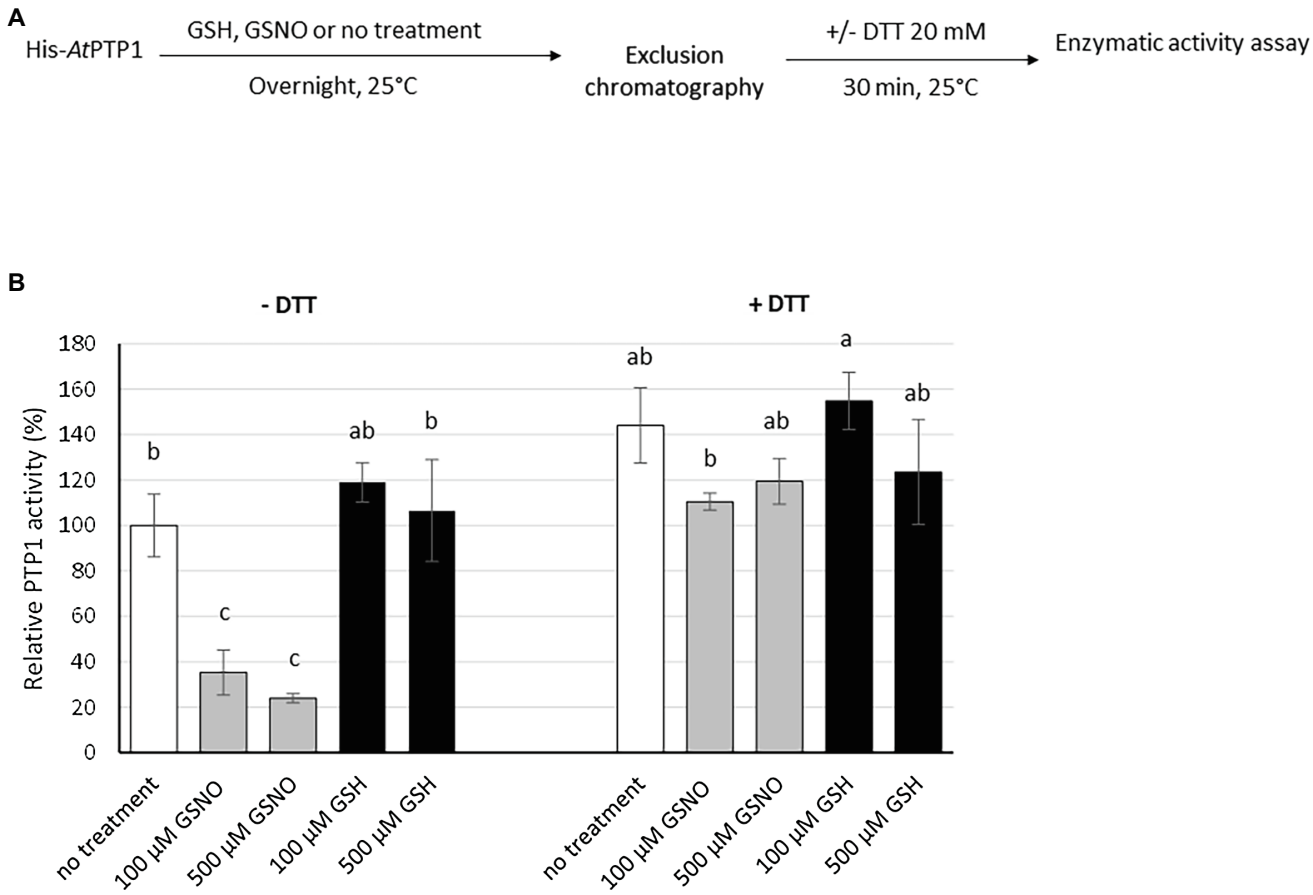
Effect of GSH or GSNO treatment on His-*At*PTP1 activity in the absence or presence of DTT. **(A)** Experimental design. **(B)** Measurement of the enzymatic rate of His-*At*PTP1 following treatment with 100 or 500 μM of GSNO (grey columns) or GSH (black columns) in the absence or presence of 20 mM DTT. Following the treatment, GSH or GSNO was removed through exclusion chromatography. Results are expressed relative to the enzyme activity measured without GSH/GSNO or DTT treatments. Values are means of four measurements ±SEM. The results shown are from one representative experiment among three independent experiments. Significant differences are denoted by different letters (*p* ≤ .05).

The phosphatase activity of His-*At*PTP1 was determined *in vitro* by spectrophotometric measurement of *p*NP (bright yellow) production from *p*NPP (colorless) as described by Montalibet et al. (2005). His-*At*PTP1 was treated overnight with 100 or 500 μM GSH or GSNO, and the enzymatic activity was measured in the absence or in the presence of 20 mM DTT (Figure 3A). The classical protocol for measuring enzymatic activity used by Montalibet et al. (2005) requires DTT in the buffer to be optimal. However, we chose to measure the activity of His-*At*PTP1 first in the absence of DTT because it breaks nitrosothiol bonds. It is therefore impossible to study the effects of S-nitrosation of the enzyme on its activity in the presence of DTT.

The left part of Figure 3B shows the rate of reaction in the absence of DTT. His-*At*PTP1 protein treated with GSNO displayed about three times less activity as compared to the GSH-treated sample, thus indicating that GSNO inhibited the enzymatic activity of His-*At*PTP1. Control experiments showed that no inhibition of activity was observed after GSH treatment, indicating that the strong inhibition observed after GSNO treatment was indeed dependent on NO itself.

Enzyme activity was then measured in the presence of DTT 20 mM (right part of Figure 3B). In the absence of any treatment, the addition of DTT increased the measurable activity of His-*At*PTP1 approximately 1.5-fold. In the presence of DTT, GSNO had no longer an inhibitory effect on His-*At*PTP1 activity. This difference indicates that the modification of His-*At*PTP1 by GSNO was reversible by the reductant.

In order to confirm the effect of S-nitrosation on the activity of the enzyme, we treated His-*At*PTP1 with SNAP, a NO donor (Supplementary Figure S2). Treatment of His-*At*PTP1 with SNAP also led to a significant decrease in its activity.

Overall, these observations suggest that the ability of GSNO or SNAP to nitrosate His-*At*PTP1 protein *in vitro* decreased the activity of the enzyme. This inhibition seems reversible as suggested by the reduced effect of GSNO in the presence of DTT during the reaction. Indeed, it can be assumed that the presence of DTT allows a break of the Cys-NO bond, thus partially restoring the activity of the enzyme. These observations suggest that NO donors modify the enzyme in a reversible manner, which impacts the activity of the His-*At*PTP1 enzyme.

### Oxidation by H_2_O_2_ Inhibits the Activity of His-*At*PTP1

**Figure 4:**
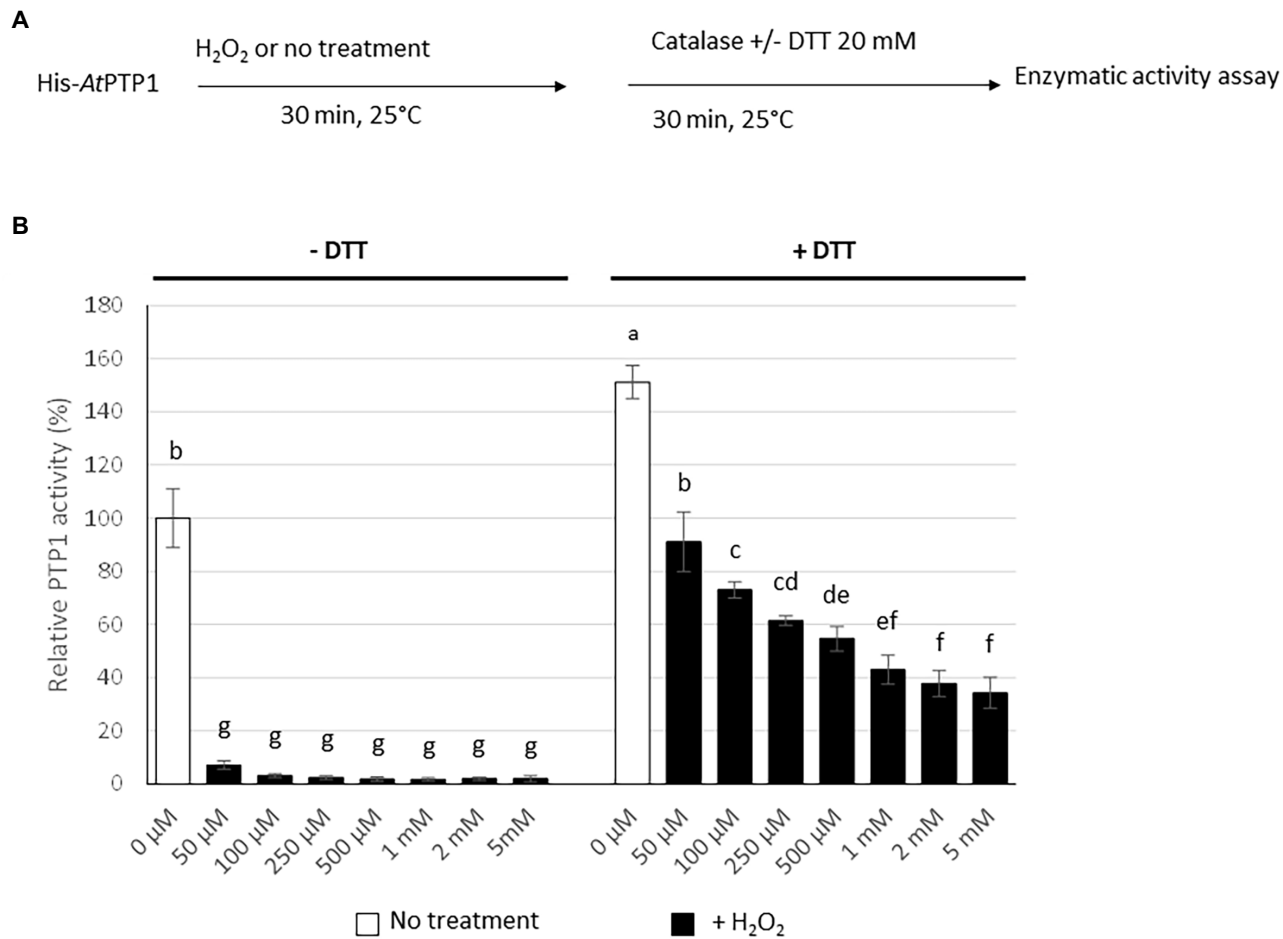
Effect of H_2_O_2_ treatment on His-*At*PTP1 activity in the absence or presence of DTT. **(A)** Experimental design. **(B)** The enzymatic activity of His-*At*PTP1 was measured following treatment with 50, 100, 250, and 500 μM or 1, 2, or 5 mM of H_2_O_2_ in the absence or presence of 20 mM DTT. Results are expressed relative to the enzyme activity measured without H_2_O_2_ or DTT treatments. Values are means of four measurements ±SEM. The results shown are from one representative experiment among three independent experiments. Significant differences are denoted by different letters (*p* ≤ .05).

The catalytic Cys residue of the human PTP1B protein can be readily oxidized *in vitro* and *in vivo*. It has been proposed that, depending on the ROS concentration, the catalytic Cys residue of PTP1B can be reversibly oxidized to sulphenic acid (Cys-SOH), but also to sulfinic acid (Cys-SO_2_H) or sulphonic acid (Cys-SO_3_H), the latter two modifications resulting in irreversible oxidation and permanent inactivation of the enzyme (Salmeen et al., 2003). His-*At*PTP1 was treated *in vitro* for 30 min with an increasing concentration of H_2_O_2_ (50 μM to 5 mM). Residual H_2_O_2_ was removed by the addition of 10 μg of catalase prior to the measurement of enzyme activity, which was performed in the absence or presence of 20 mM DTT which eliminates the reversible modification of His-*At*PTP1 (Figure 4A). A drastic reduction of His-*At*PTP1 activity was observed after H_2_O_2_ treatment in the absence of DTT. This phenomenon is visible as early as the 50 μM concentration (Figure 4B, left part). This condition allows visualizing both reversible and irreversible oxidation of His-*At*PTP1. In contrast, enzyme activity was partially restored after the addition of DTT (Figure 4B, right part), revealing the reversible character of some Cys oxidations. The presence of DTT eliminates the reversible modification of His-*At*PTP1. This suggests that the proportion of irreversible oxidation increases with H_2_O_2_ concentration, a dose-dependent residual inactivation being observed with DTT.

### Prior S-Nitrosation of His-*At*PTP1 Protects It From Irreversible Oxidation by H_2_O_2_

**Figure 5:**
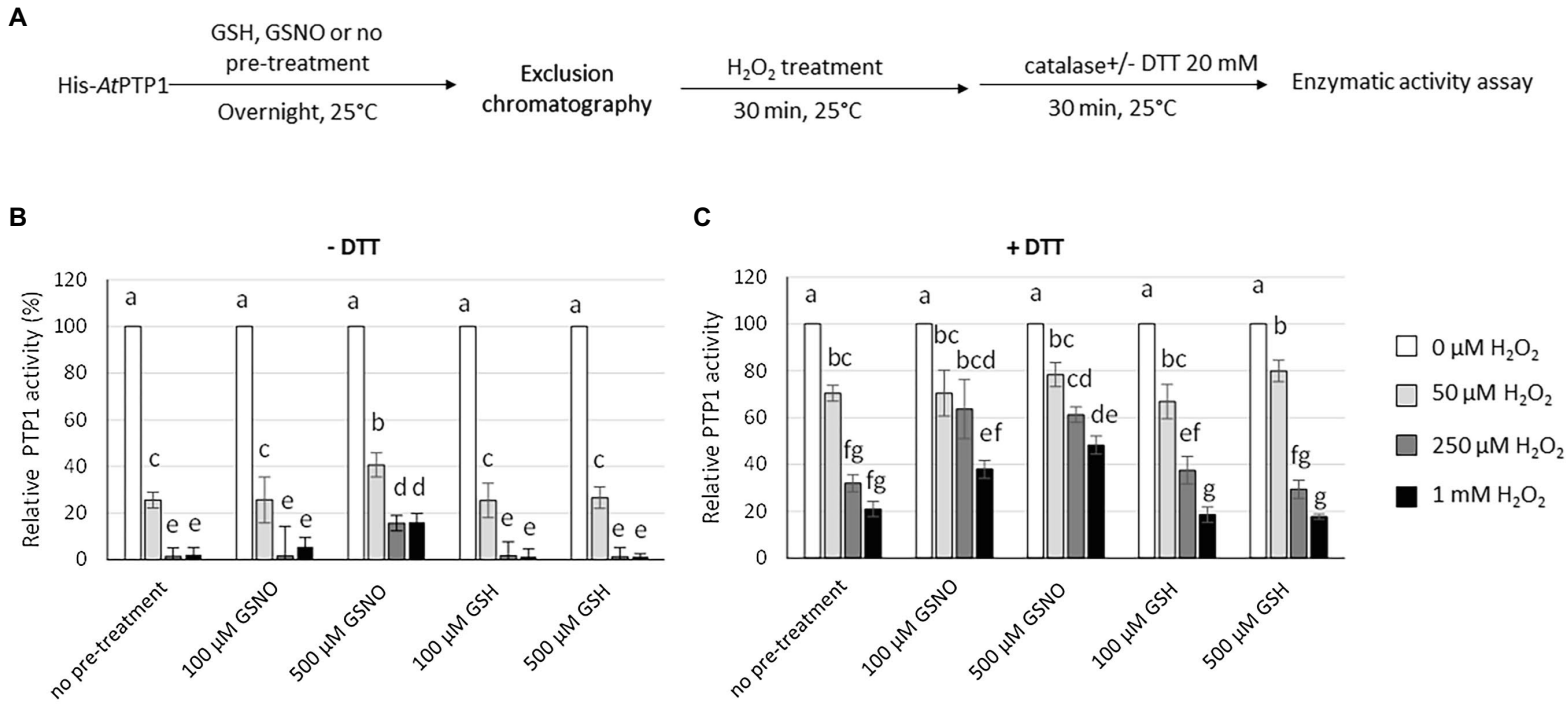
Effect of H_2_O_2_ treatment on His-*At*PTP1 activity in the absence or presence of DTT following pre-treatment with GSH or GSNO. **(A)** Experimental design. **(B)** Measurement of the enzymatic rate of His-*At*PTP1 following treatment with 50 and 250 μM or 1 mM of H_2_O_2_ in the absence of 20 mM DTT after pre-treatment with 100 or 500 μM of GSH or GSNO. **(C)** Measurement of the enzymatic rate of His-*At*PTP1 following treatment with 50 μM, 250 μM or 1 mM of H_2_O_2_ in the presence of 20 mM DTT after pre-treatment with 100 or 500 μM of GSH or GSNO. Values are means of four measurements ±SEM. The results shown are from one representative experiment among three independent experiments. Significant differences are denoted by different letters (*p* ≤ .05).

To test the protective effect of S-nitrosation against irreversible oxidation of His-*At*PTP1, we incubated it overnight with 100 or 500 μM GSH or GSNO in the absence or presence of 20 mM DTT. To avoid a potential interaction between GSH/GSNO and H_2_O_2_, we purified the protein on a G50 column to remove GSH/GSNO after the pre-treatment. His-*At*PTP1 was then treated with an increasing concentration of H_2_O_2_ (between 50 μM and 1 mM) subsequently degraded by 10 μg of catalase before measuring the activity in the absence or presence of DTT (Figure 5A). We then measured the reversible and irreversible oxidation of His-*At*PTP1 with or without prior S-nitrosation in the absence (Figure 5B) or in the presence of DTT (Figure 5C). As reported in Figure 4, in the absence of GSNO pre-treatment, a dose-dependent inhibitory effect of H_2_O_2_ on His-*At*PTP1 activity was observed, this effect being stronger in the absence (Figure 5B) than in the presence (Figure 5C) of DTT. After pretreatment of the enzyme with 500 μM of GSNO, the effect of H_2_O_2_ treatment on its activity in the absence of DTT was reduced compared to pretreatment with GSH. Indeed, the activity measured in response to 1 mM H_2_O_2_ after pre-treatment with 500 μM GSNO was increased 7.33- and 10.8-fold following no pre-treatment or pre-treatment with 500 μM GSH, respectively (Figure 5B). The effect of GSNO was diminished following the use of DTT as it decreases to factors of 2.4 and 2.7 following no pre-treatment or pre-treatment with GSH, respectively (Figure 5C). S-nitrosation of His-*At*PTP1 could therefore protect the enzyme from reversible and irreversible oxidation. As shown above, S-nitrosation by GSNO and part of the oxidation of His-*At*PTP1 are reversible following DTT treatment. We assume that in the presence of DTT, we observed only the inhibitory effect due to the irreversible oxidation (Figure 5C). The decrease in His-*At*PTP1 activity was less pronounced when the enzyme was pretreated with 500 μM GSNO than with GSH. Thus, the modification of His-*At*PTP1 by GSNO might protect it from irreversible oxidation by H_2_O_2_. This observation agrees with the mass spectrometry results of Chen et al. (2008) showing that pre-treatment of PTP1B recombinant protein with the NO donor SNAP prevents its irreversible oxidation. To rule out the possibility that the oxidation protection effect was due to glutathionylation as in the case of the *Gm*PTP protein, we performed the same experiment as before but use the NO donor SNAP (1 mM) instead of GSNO. The data presented in Supplementary Figure S3 show that the use of SNAP reduced the H_2_O_2_-induced inhibition of His-*At*PTP1 activity by about 15.5-fold in the absence of DTT and by 4.5-fold in the presence of DTT. We confirm that S-nitrosation would protect the protein from irreversible oxidation by H_2_O_2_.

## Discussion

After production and purification of the His-tagged *At*PTP1 protein, we showed its S-nitrosation *in vitro* in response to the trans-nitrosylating agent GSNO. Our results suggest that the catalytic cysteine C265 is the major site of S-nitrosation on His-*At*PTP1. An increase in the rate of S-nitrosation was however detected for other Cys residues but the data are not significantly different between GSH and GSNO treatments. Two studies have shown by mass spectrometry (ESI-MS) that the human PTP1B protein is a target of S-nitrosation (Chen et al., 2007, 2008). In particular, treatment of PTP1B with the NO donor SNAP led to the formation of S-nitrosothiol at C215. S-nitrosation occurs most readily at the catalytic Cys residue but two other residues, C32 and C92, were also susceptible to S-nitrosation. As these two residues are on the surface of the protein, the authors deduced that their S-nitrosation could be aspecific because of an easier access for the NO donor (Chen et al., 2008).

We next analyzed the direct effects of NO exogenous treatment on the enzymatic activity of His-*At*PTP1 by measuring the colorimetric transformation of *p*NPP to *p*NP. It appears that artificially generated NO from GSNO or SNAP inhibited the activity of the protein. This process was reversible since treatment with DTT restored the activity of the enzyme. The activity of PTP1B protein on cell lysates or intact A431 or Jurkat cells was also rapidly inhibited by the addition of nitrosothiols, with the addition of DTT removing the inhibition (Li and Whorton, 2003). Moreover, using in-gel phosphatase activity assay, Chen et al. (2007) were able to prove that treatment with increasing concentrations of SNAP led to the appearance of an inactivated form of PTP1B. Thus, it appears that both *At*PTP1 and PTP1B protein activities are inhibited by NO.

Oxidation of *At*PTP1 by H_2_O_2_ also inhibited its activity, but unlike treatment with GSNO, this process was not completely restored following subsequent treatment with DTT. This regulatory mechanism has also been demonstrated for PTP1B (Salmeen et al., 2003; van Montfort et al., 2003). The inactivation of PTP1B was not prevented in the presence of reducing agents, but just slowed down because of the faster reaction of H_2_O_2_ with the enzyme as compared to DTT (Denu and Tanner, 1998). However, once oxidized by H_2_O_2_ to PTP1B-SOH, its activity can be reactivated by reduction with DTT, GSH, β-mercaptoethanol, or Cys (Denu and Tanner, 1998). Therefore, the activities of *At*PTP1 and PTP1B undergo similar modes of regulation following H_2_O_2_ treatment.

We finally studied the effect of H_2_O_2_ on *At*PTP1 activity and the impact of prior S-nitrosation on this process. It appeared that a pre-treatment with GSNO or SNAP prevented the irreversible oxidation of *At*PTP1 by H_2_O_2_. Although we cannot totally exclude the protection of C165 from oxidation by glutathionylation as shown for *Gm*PTP, our results show that this process would be rather provided by S-nitrosation. In addition, the mechanism would anyway be different from the one described for GmPTP as the C78 residue involved in the disulfide bridge formation with the catalytic Cys residue is not conserved in *A. thaliana*. However, the protective effect of NO against *At*PTP1 oxidation that we measured *in vitro* needs to be verified *in vivo*. On the one hand, the effects of exogenous treatments with NO donors or H_2_O_2_, or endogenously in response to stress, could perhaps be detected by mass spectrometry at the catalytic Cys residue. On the other hand, it is complicated to consider measuring an effect on protein activity given the number of proteins capable of hydrolyzing *p*NPP to *p*NP. Previously, Chen et al. (2008) highlighted an increased cellular level of NO in COS7 cells, due to pre-treatment with a NO donor or to ectopically activated NOS expression. Such NO increase inhibited ROS-induced irreversible oxidation of the endogenous PTP1B protein. The authors of this study indicated that the data obtained *in vitro* could be verified *in vivo* to analyze the redox status of endogenous PTPases in response to a stimulus that induces NO and/or ROS production in cells. They suggested to immunoprecipitate the PTPase of interest from a cell lysate thanks to specific antibodies and then to identify the S-nitrosated residues by nanoLC-nano-ESI-MS/MS analyses. This method could be used to demonstrate that *At*PTP1 is regulated by S-nitrosation *in vivo*. However, such specific antibodies are not yet available.

Protection from irreversible oxidation by S-nitrosation is not unique to PTPases as other proteins such as galectin-2 are regulated in this way. Galectin-2 are soluble animal lectins defined by their conserved carbohydrate recognition domain and their affinity for β-galactosides. Mouse galectin-2 (mGal-2) which is mainly expressed in the gastrointestinal tract is regulated by oxidation and S-nitrosation. Oxidation of Cys 57 of mGal-2 by H_2_O_2_ results in the loss of its sugar-binding capacity and thus its inactivation. In contrast, pretreatment of mGal-2 with S-nitrosocysteine, an S-nitrosating agent, has no effect on its sugar-binding capacity but prevents H_2_O_2_-induced inactivation (Tamura et al., 2015, 2016). It appears that S-nitrosation slows down the H_2_O_2_-induced aggregation of mGal-2 by stabilization of the hydrophobic pocket around Cys 57 (Sakakura et al., 2018). Understanding the molecular mechanisms of protection of PTP1B or *At*PTP1 by S-nitrosation against oxidative inactivation may provide a better understanding of the protective function of PTPases in response to oxidative stress.

Under standard culture conditions, Bartels et al. (2009) did not identify any macroscopic effects of the mutation in the *AtPTP1* gene when measuring leaf mass of 21-day-old null mutant plants, which would indicate that it is primarily *At*MKP1 that has a role in Arabidopsis. In contrast, the *mkp1ptp1* double mutant has a stronger phenotype than the *mkp1* mutant. *At*PTP1 could therefore have a complementary role to that of *At*MPK1, especially since *At*PTP1 and *At*MKP1 display different cellular localizations. The nuclear localization of *At*PTP1 could especially give it a specific regulatory role. Only few data are available regarding the regulation of *At*MKP1. It can be phosphorylated at Ser and Thr residues by *At*MPK6 but its sensitivity to oxidation or S-nitrosation has not been studied. The possibility of regulating *At*PTP1 activity by oxidation and/or S-nitrosation could allow a finer adjustment in response to abiotic stress. Again, if a specific antibody would be available to immunoprecipitate *At*PTP1, it would be relevant to study post-translational modifications of the enzyme in response to (a)biotic stresses.

Overall, our data showed that the *Arabidopsis thaliana* PTP1 protein has a similar regulation of its activity as other PTPases in response to oxidative stress. S-nitrosation of PTPase proteins is likely to be a general mechanism for preventing the permanent inactivation of these enzymes caused by oxidative stress.

## Data Availability Statement

The datasets presented in this study can be found in online repositories. The names of the repository/repositories and accession number(s) can be found at: ProteomeXchange, PXD030071.

## Author Contributions

AB-B, DW, and VN-F supervised the research and wrote the manuscript. JR and VN-F designed the experiments. AK, CR, JR, and VN-F carried out His-*At*PTP1 production and purification. JR, VN-F, and SH measured the enzymatic activities of His-*At*PTP1. CP analyzed the samples by mass spectrometry. JA performed sequence alignments of the different PTP1s. All authors have read and approved the final manuscript.

## Funding

Work in our lab is supported by the Ministère de l’Enseignement Supérieur, de la Recherche et de l’Innovation, Investissements d’Avenir program, project ISITE-BFC NOISELESS (contract ANR-15-IDEX-0003; grant NOISELESS-RA18041.AEC.IS), the Agence Nationale de la Recherche, project ALGAE-NOS (grant ANR-18-CE20-0022-02), and the Région de Bourgogne, project RHINO (grant 2018Y-07113).

## Conflict of Interest

The authors declare that the research was conducted in the absence of any commercial or financial relationships that could be construed as a potential conflict of interest.

## Publisher’s Note

All claims expressed in this article are solely those of the authors and do not necessarily represent those of their affiliated organizations, or those of the publisher, the editors and the reviewers. Any product that may be evaluated in this article, or claim that may be made by its manufacturer, is not guaranteed or endorsed by the publisher.

## Footnotes

^1^ http://proline.profiproteomics.fr/
