# Supplementary figures and images for: S-Nitrosation of Arabidopsis thaliana Protein Tyrosine Phosphatase 1 Prevents Its Irreversible Oxidation by Hydrogen Peroxide
Source: Front Plant Sci. 2022 Feb 11;13:807249. doi: 10.3389/fpls.2022.807249 (PMC8867174; doi:10.3389/fpls.2022.807249)

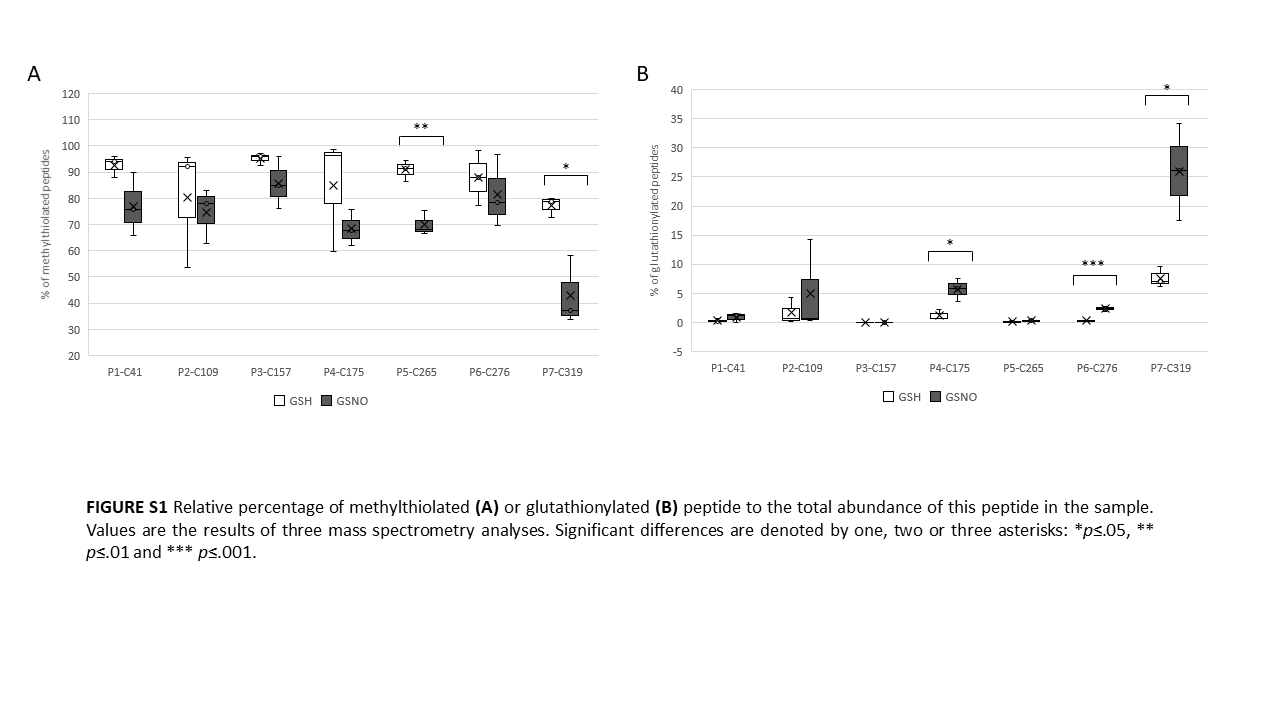

Supplement: Supplementary file 1 [file Image_1.tif]

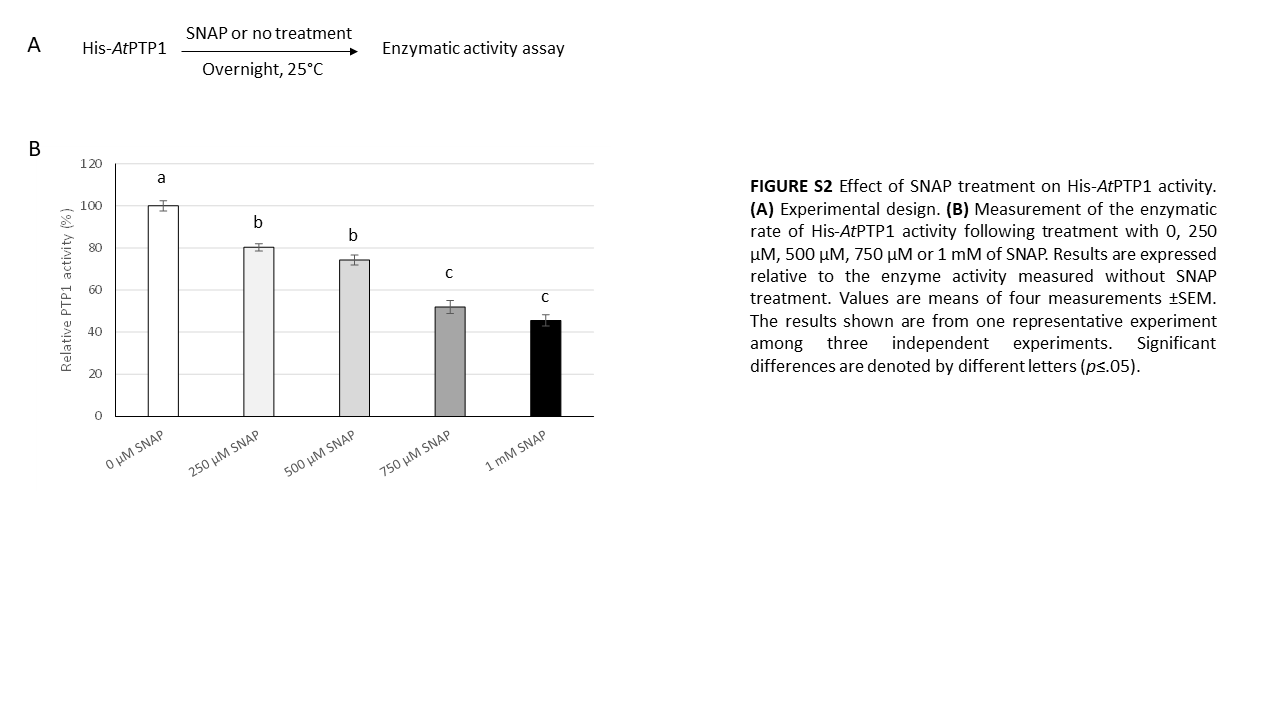

Supplement: Supplementary file 2 [file Image_2.tif]

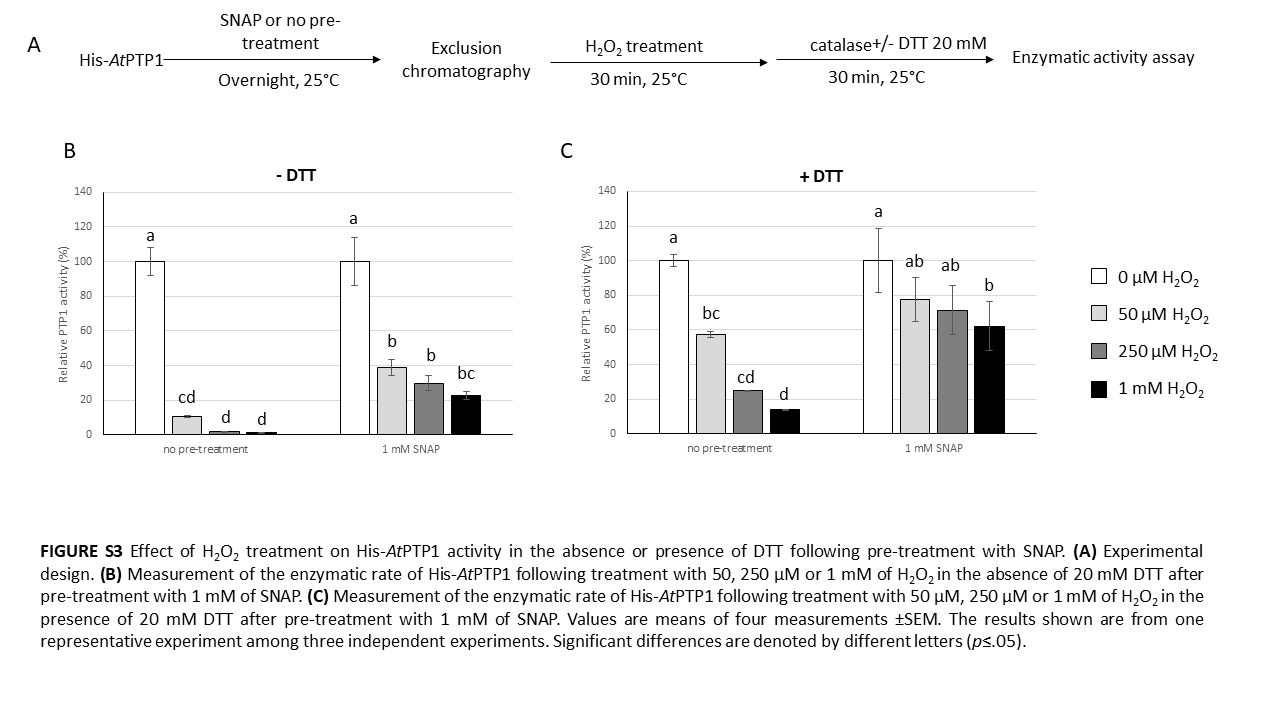

Supplement: Supplementary file 3 [file Image_3.tif]
